# Transcriptomic Analysis on Responses of Murine Lungs to *Pasteurella multocida* Infection

**DOI:** 10.3389/fcimb.2017.00251

**Published:** 2017-06-20

**Authors:** Chenlu Wu, Xiaobin Qin, Pan Li, Tingting Pan, Wenkai Ren, Nengzhang Li, Yuanyi Peng

**Affiliations:** ^1^College of Animal Science and Technology, Southwest UniversityChongqing, China; ^2^Key Laboratory of Agro-ecological Processes in Subtropical Region, Scientific Observing and Experimental Station of Animal Nutrition and Feed Science in South-Central, Ministry of Agriculture, Hunan Provincial Engineering Research Center of Healthy Livestock, Institute of Subtropical Agriculture, Chinese Academy of SciencesChangsha, China

**Keywords:** *P. multocida*, lung, IFN-γ, IL-17, transcriptomic analysis

## Abstract

*Pasteurella multocida* infection in cattle causes serious epidemic diseases and leads to great economic losses in livestock industry; however, little is known about the interaction between host and *P. multocida* in the lungs. To explore a fully insight into the host responses in the lungs during *P. multocida* infection, a mouse model of *Pasteurella* pneumonia was established by intraperitoneal infection, and then transcriptomic analysis of infected lungs was performed. *P. multocida* localized and grew in murine lungs, and induced inflammation in the lungs, as well as mice death. With transcriptomic analysis, approximately 10^7^ clean reads were acquired. 4236 differently expressed genes (DEGs) were detected during *P. multocida* infection, of which 1924 DEGs were up-regulated. By gene ontology (GO) and Kyoto encyclopedia of genes and genomes (KEGG) enrichments, 5,303 GO enrichments and 116 KEGG pathways were significantly enriched in the context of *P. multocida* infection. Interestingly, genes related to immune responses, such as pattern recognition receptors (PRRs), chemokines and inflammatory cytokines, were significantly up-regulated, suggesting the key roles of these genes in *P. multocida* infection. Transcriptomic data showed that IFN-γ/IL-17-related genes were increased, which were validated by qRT-PCR, ELISA, and immunoblotting. Our study characterized the transcriptomic profile of the lungs in mice upon *Pasteurella* infection, and our findings could provide valuable information with respect to better understanding the responses in mice during *P. multocida* infection.

## Introduction

*Pasteurella multocida* is a gram-negative facultative anaerobic bacillus, which infects a wide range of domestic and wild animals, mainly causing hemorrhagic septicemia and respiratory diseases. *P. multocida* is classified into five capsular serogroups (A, B, D, E, and F) based on specificity of capsular antigens, and 16 serotypes based on lipopolysaccharide (LPS) antigens (Kubera et al., 2017). In China, *P. multocida* capsular serogroup B induces hemorrhagic septicemia in cattle, while *P. multocida* capsular serogroup A is mainly associated with pulmonary infection, which causes economic losses in cattle (Dabo et al., 2007). *P. multocida* serogroup A is one of nasopharyngeal commensal pathogens associated with respiratory diseases in livestock. During the alteration of external environment (e.g., long-distance transport, weather variations) or decrease in host immunity, *P. multocida* will be extended from the upper respiratory tract to enter the lower respiratory tract, causing pneumonia in cattle. The virulence factors (e.g., the capsule, fimbriae, adhesion proteins, toxins, iron regulation and iron acquisition proteins and outer membrane proteins) of *P. multocida* are thought to evade immune defenses in cattle, which may result in widespread existence of pneumonic pasteurellosis (Harper et al., 2006; Griffin et al., 2010).

Currently, the vaccine is the main method of preventing pneumonic pasteurellosis. However, complete protection of vaccine against *P. multocida* infection was not available, which may be due to our inadequate understanding *P. multocida-*host interaction and limited recognition of immune responses against *P. multocida* infection in host (Mathy et al., 2002). Recently, it has been reported that the responses in the lungs after *P. multocida* infection mainly include expression of some inflammatory mediators and cell apoptosis in mice or cattle. For example, *P. multocida* infection with intratracheal inoculation induces the mRNA expressions of pro-inflammatory cytokines (e.g., *Tnf-*α*, Il-6, Il-1, Il-8*, and *Il-12*), and the increase in the number of neutrophils in the lungs of cattle (Mathy et al., 2002). Although we have the understanding about the expressions of these cytokines and the accumulation of neutrophils in the lungs, a comprehensive immune responses (e.g., Th17 responses) in host against *P. multocida* remains to be explored. In addition, the alterations of other aspects (e.g., metabolic responses) in the lungs during *P. multocida* infection are still unclear. Transcriptomic sequencing has been applied to provide the new insights into molecular mechanisms and to explore the interaction between host and pathogens, such as mango-*Fusarium mangiferae*, tilapia-*Streptococcus agalactiae*, and epithelial-pneumococcal interaction (Aprianto et al., 2016; Liu et al., 2016; Wang et al., 2016). Thus, to explore the host responses during *P. multocida* infection, this study firstly established a mouse model of *Pasteurella* pneumonia using *P. multocida* serotype A strain CQ2. Subsequently, comprehensive gene expression profiles of infected and sham-infected lungs were explored using RNA-seq analysis. Differently expressed genes (DEGs) were acquired to for functional annotations and classifications, and some DEGs were validated using qRT-PCR, ELISA, and immunoblotting. Mice were selected as models for this study because it is difficult to use the cattle in research considering the high economic cost with the cattle. Also, there are various similarities in pathogenesis between mice and cattle after *P. multocida* infection, thus the mice are selected to act as substitutable models of cattle in many studies. For example, similar to cattle, *P. multocida* infection in mice by an intraperitoneal route or an intranasal route also results up-regulated pro-inflammatory cytokines, reactive oxygen species (ROS) and lymphocyte apoptosis (Praveena et al., 2010; Wu et al., 2014). This study revealed transcriptomic profile of murine lungs upon *Pasteurella* infection and provided a new insight into interaction between mice and *P. multocida* using transcriptomic analysis.

## Materials and methods

### Culture of bacteria

This study used *P. multocida* serotype A strain CQ2 (PmCQ2, GenBank accession number: LIUN00000000), which is a highly virulent strain and isolated from a bovine pneumonic lung, causing severe pulmonary inflammation and high mortality in mice (Li et al., 2016). As previously described, PmCQ2 was routinely cultured in Martin's broth agar containing 5% horse serum (Ren et al., 2013a; Chen et al., 2014).

### Mouse infection

All animal studies were performed according to the guidelines of the Laboratory Animal Ethical Commission of the Southwest University, and the procedures used in this study were approved by the Laboratory Animal Ethical Commission. Six to eight week-old, female C57BL/6 mice (weight 18–22 g) were brought from Laboratory Animal Center of Third Military Medical University (Chongqing, China). The mice were housed in the individual ventilated cages (experimental animals IVC factory, Suzhou, China), keeping temperature at 20–30°C relative humidity at 50–60% and lighting cycle at 12 h/day. Total 130 mice were used in this study. In the infected group, the mice were challenged by an intraperitoneal injection of PmCQ2 at the dose of 2.2 × 10^5^ (LD_50_) colony-forming units (CFU) in 100 μL. In the control group, mice (gender and age matched) were injected intraperitoneally with equal dose of saline. Survival rates (*n* = 10 in each group) were measured in both groups after injection. Mice were also euthanized to collect lung tissues at 8, 16, and 24 h post-infection. Serum samples were also collected after infection.

### Bacterial colonization

To measure the bacterial load, the lung tissues (*n* = 10 in each group) were collected at 0, 8, 16, and 24 h post-bacterial infection. The tissues were homogenized aseptically and bacterial loads were quantified by 10-fold serial dilution in saline. These different dilutions were plated in triplicate on Martin's broth agar and were incubated at 37°C up to 24 h to count CFU.

### Histopathological examination and FISH

For histopathological examination, the lung tissues (*n* = 6 in each group) were immediately fixed in the 4% paraformaldehyde for 24 h, dehydrated in graded ethanol, and then embedded in paraffin wax. The tissues were sliced at 3 μm thick and then stained with hematoxylin and eosin (H&E). Histopathological scoring was performed by a pathologist blinded to treatment and control groups, and the scoring was mainly based on interstitial inflammation, vascular endothelialitis, bronchitis, edema, serous effusion and thrombus formation (van den Boogaard et al., 2015). All parameters were scored separately from 0 (lesion absent) to 3 (severe lesion) (Praveena et al., 2014; Table 1).

**Table 1:** The criteria of histopathological scores.

**Histopathological scores**	**Inflammatory cells**	**Alveolar/bronchial epithelium**	**Blood vessels**	**Inflammatory exudation**
0 (no lesion)	Not detected	Normal	Normal	Not dected
1 (mild)	A small amount of inflammatory cell infiltration (<10 cells in a field of vision)	Slight degeneration and swelling in alveolar/bronchial epithelial cells	Slight congestion	A small amount of serous exudate
2 (moderate)	Moderate inflammatory cell infiltration (10–20 cells in a field of vision)	Moderate degeneration and swelling in alveolar/bronchial epithelial cells	Moderate congestion or hemorrhage; vascular endothelial swelling	Moderate serous exudate
3 (severe)	A lot of inflammatory cell infiltration (>20 cells in a field of vision)	Epithelial cells appear serious degeneration, swelling or even necrosis, shedding	Severe bleeding; plenty of red blood cells in the alveolar cavity; vascular interstitial space widened	A large amount of serous exudate

For fluorescence *in situ* hybridization (FISH), the tissues (*n* = 6 in each group) were fixed in the 4% paraformaldehyde for 1 h. Other steps about paraffin section processing were same as descripted above. Then sections were denatured in degeneration buffer [70% formamide, 30% 2 × saline-sodium citrate (2 × SSC)] at 78°C for 8 min and the probes were denatured in hybridization buffer (0.9 M NaCl, 0.1% SDS, 100 mM Tris PH 7.2, 15% formamide, 10% SDS), followed by hybridization overnight at 42°C. 20 μL hybridization buffer containing 15 μg probes were applied to per section. This study used pmhyb449, 59-CTATTTAACAACATCCCTTC-39 (S-S-Pmul-0449-a-A-20) (Sangon Biotech, China) to detect PmCQ2 (Mbuthia et al., 2001). The probes were labeled with Cy3. After washed by wash buffer (50% formamide, 50% 2 × SSC) for three times with 5 min in each time, cell nuclei were determined by counterstained with 4′, 6-diamidino-2-phenylindole (DAPI, Beyotime Biotech, China) to evaluate cellular morphology. Fluorescence was detected using fluorescence microscope (Olympus). Six mice were included in each group and three slices were conducted in each murine lung. The strength of the fluorescent signals was quantitatively analyzed in three visions per slice.

### RNA isolation, cDNA library construction and illumina deep sequencing

Total RNA from approximately 150 mg lung tissues (*n* = 3 in each group) was extracted using Trizol reagent (Invitrogen, USA) following the manufacturer's protocol. Integrity of RNA was confirmed by 2100 Bioanalyzer (Agilent Technologies). The samples for transcriptomic analysis were prepared using Illumina's kit following manufacturer's recommendations. Briefly, mRNA was purified using oligo (dT) magnetic beads. By using the fragmentation buffer, the mRNA was fragmented into short fragments (about 200 bp), and then the first strand of cDNA was synthesized with random hexamer-primer using the mRNA fragments as templates. Buffer, dNTPs, RNase H, and DNA polymerase I were added to synthesize the second strand. The double-stranded cDNAs were purified with QiaQuick PCR extraction kit (Qiagen) and eluted with elution buffer for end repair and poly (A) addition. Finally, sequencing adapters were ligated to the 5′ and 3′ ends of the fragments. The fragments were purified by agarose gel electrophoresis and enriched by PCR amplification to create a cDNA library. The cDNA library was sequenced on the Illumina sequencing platform (HiSeqTM 2000) and 100 bp paired-end reads were generated.

### Functional annotation of DEGs

Raw data were firstly processed using the NGS QC Toolkit. In this step, clean data (clean reads) were obtained by removing reads containing adapter, reads containing ploy-N and low quality reads from raw data. All the downstream analyses were based on clean data with high quality. The clean reads were mapped to reference genome (ftp://ftp.ensembl.org/pub/release-84/fasta/mus_musculus/dna/) and reference transcript (ftp://ftp.ensembl.org/pub/release-84/fasta/mus_musculus/cdna/Mus_musculus.GRCm38.cdna.all.fa.gz) using bowtie2 or Tophat (http://tophat.cbcb.umd.edu/) with default parameters by slightly modified (Langmead and Salzberg, 2012; Kim et al., 2013). Transcriptomic sequencing quality was displayed by mapping ratio to the reference genome and transcriptome. Based on results of clean reads mapping to reference genome, fragments per kilo base of exon per million fragments mapped (FPKM) values of genes were calculated and count value were calculated using eXpress (Mortazavi et al., 2008).

The read counts of each gene were obtained by htseq-count (Anders et al., 2015). Differential expression analysis was performed using estimateSizeFactors in DESeq (2012) R package, and p value and fold-change (FC) value were identified using nbinomTest in DESeq (2012) R package. *P* < 0.05 was set as the threshold for significantly differential expression. Hierarchical cluster analysis is used to identify differentially expressed genes with certain patterns of expression under two different salinity challenge using R. The settings for the calculations were as follows: similarity was measured by euclidean, and clustering method was complete linkage. GO and KEGG pathway enrichment analysis of the DEGs were performed using R based on hypergeometric distribution.

All the data discussed in this study have been deposited to NCBI's Gene Expression Omnibus (GEO) database and the accession number is SRR5186264.

### Quantitative real-time RT-PCR (qRT-PCR)

Total RNA of the lungs (*n* = 6 in each group) were acquired as described previously (Shi et al., 2015). cDNA was synthesized using a PrimeScript™ RT reagent Kit with gDNA Eraser (TaKaRa, Dalian, China). qRT-PCR was performed according to previous study (Ren et al., 2013a). Beta-actin was used as a reference gene to normalize transcript levels of target genes. Specific primers were designed according to the reference sequences in NCBI with Primer 5.0 software and the primer sequences were listed in Supplementary Table 1. The relative expression levels of genes were acted as a ratio of the target gene to the control gene using formula 2-^(ΔΔCt)^, where ΔΔCt = (Ct for a target gene − Ct for the β-actin gene) in a treatment group − (Ct for a target gene − Ct for the β-actin gene) in the control (Ren et al., 2013b).

### Immunoblotting analysis

Immunoblotting analysis was performed as described previously (Kamat et al., 2013). Briefly, approximate 100 mg frozen lung tissue powder (*n* = 5 in each group) was lysed in 1 mL RIPA buffer containing 100 mM PMSF to extract pulmonary total proteins. The protein concentration was determined using a bicinchoninic acid (BCA) protein assay kit (Beyotime, China). Equal amount of proteins (10 μg) were separated by 10% SDS-PAGE and transferred onto PVDF membrane (BioRad). The blots were blocked with 5% (w/v) skimmed milk in TBST (50 mM Tris–HCl, 150 mM NaCl, 0.1% Tween- 20, pH 7.5) for 2 h. Then blots were incubated with primary antibodies overnight at 4°C. Subsequently blots were incubated with the horseradish peroxidase (HRP)-conjugated secondary antibodies for 1 h at 37°C. After ECL substrates were added, the blots were analyzed using light imaging system (Tanon 5200, China). Before each step, the blots were washed five times for 5 min with TBST.

In this study, β-actin (Proteintech) was determined as an internal control of the western blot. The primary antibodies were activator of transcription 1 (STAT1) (Beyotime Biotech, China), STAT3 (Wanleibio, China), p-STAT1 Y701 (Cell Signaling Technology) and p-STAT3 Y705 (Cell Signaling Technology). All secondary antibodies were peroxidase-conjugated affinipure goat anti-rabbit IgG (Proteintech).

### Enzyme linked immunosorbent assay (ELISA)

Lung homogenates (*n* = 6 in each group) were freezing and thawing (frozen in liquid nitrogen for 5 min and then melted on ice) three times. After centrifugation at 12,000 rpm for 10 min at 4°C, supernatant were acquired. Cytokines (IL-17, IL-6, and IFN-γ) were detected in the supernatant or the serum with ELISA kits in accordance with the manufacturer's protocol. ELISA kit for IFN-γ detection was purchased from Cusabio (Wuhan, China). Other cytokine ELISA kits were purchased from eBioscience, USA.

### Statistical analysis

All data were expressed as means ± Standard Error of Mean (SEM). All statistical analyses were performed using GraphPad Prism software. Survival rates of mice were evaluated using Kaplan-Meier analysis (Prism 6.0). Data between groups in H&E experiments were analyzed by Mann-Whitney U tests (Prism 6.0). All the other data between two groups were evaluated using unpaired, two-tailed Student's *t*-test (Prism 6.0). Data among more than two groups were analyzed by the one-way ANOVA followed by Dunnett multiple comparisons (Prism 6.0). Significant differences were considered at *p* < 0.05. ^*^means *p* < 0.05, ^**^means *p* < 0.01, ^***^means *p* < 0.001.

## Results

### *P. multocida* infection in mice

**Figure 1:**
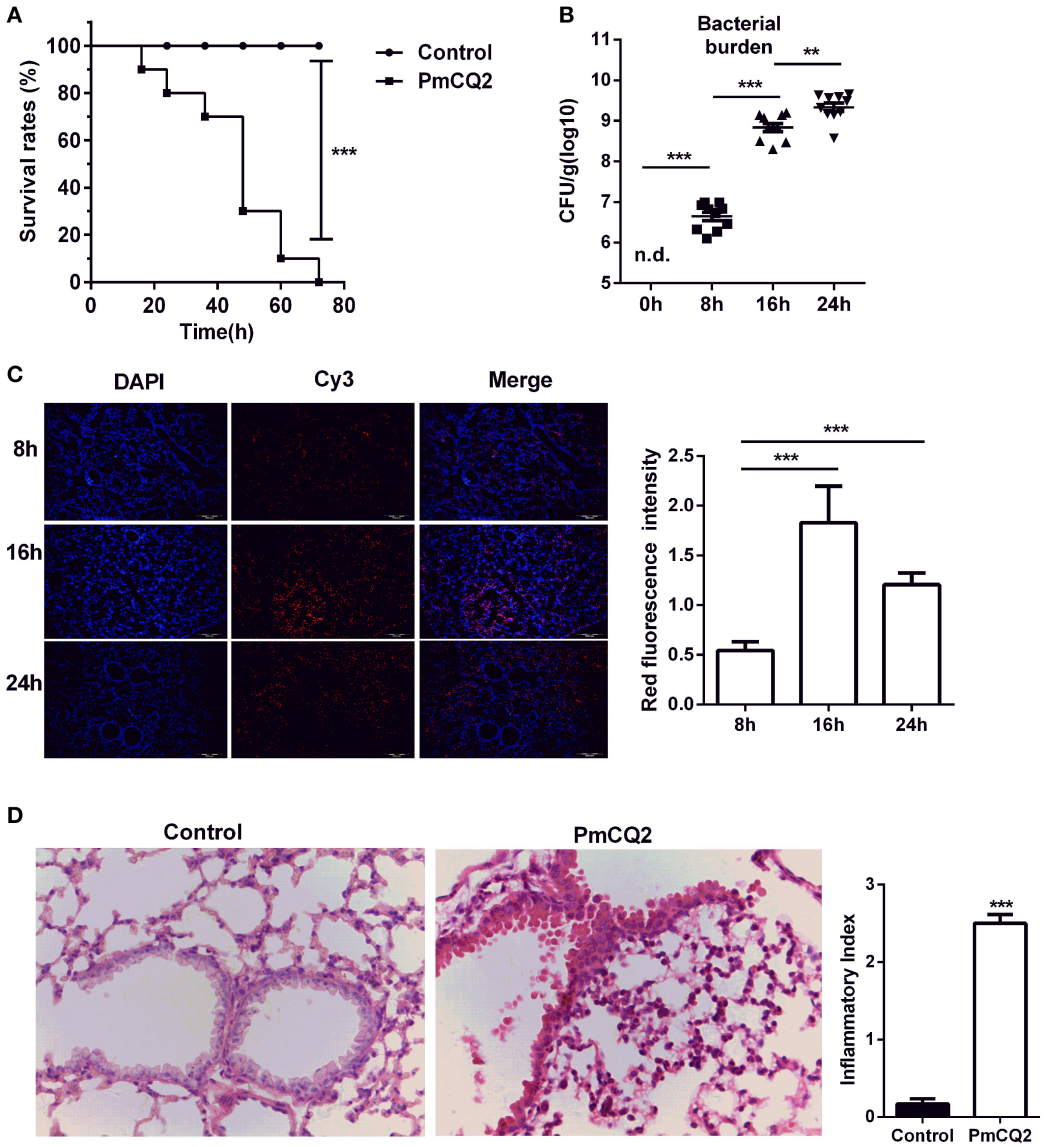
A mouse model of *Pasteurella* pneumonia caused by PmCQ2. **(A)** A high mortality rate in mice caused by PmCQ2. (*n* = 10, Kaplan-Meier analysis). **(B)** The proliferation of bacteria in the lung tissues (*n* = 10, one-way ANOVA). **(C)** The specific distribution of PmCQ2 in the lung tissues via FISH (*n* = 6, one-way ANOVA). **(D)** The severe pulmonary inflammatory lesions caused by PmCQ2 with H&E staining (*n* = 6, Mann-Whitney U tests, magnification 400X). Each point represents an individual mouse in the graph **(B)** and the data was expressed as means ± SEM. ^**^P < 0.01, ^***^P < 0.001.

After infection, the survival rates were analyzed. There was a significant difference between infected group and control group (Figure 1A). All mice (*n* = 10) rapidly approach moribund within 3 days post-infection, whereas, no mortality was observed in control group. Then, bacterial load was counted in murine lungs at 0, 8, 16, 24 h post-infection (hpi). Although, PmCQ2 could not be detected in healthy murine lungs, the bacterial load was approximately 10^6^ CFU/g at 8 hpi, and grew significantly at 16 and 24 hpi (Figure 1B). Also, FISH with specific probe of PmCQ2 (pmhyb449) showed the presence of PmCQ2 in the infected murine lungs (Figure 1C). PmCQ2 was detected widely in the alveolar walls, terminal bronchioles, and interstitial areas. At 16 and 24 hpi, bacterial burden was significantly higher than those at 8 hpi, whereas, there was no significant difference about the bacterial load between 16 and 24 hpi. The pulmonary lesions were also explored by H&E staining. After infection with PmCQ2, there were more severe inflammatory lesions (Figure 1D). The numbers of inflammatory cells in the peribronchial, alveolar and perivascular area of infected group were significantly higher than those in the control group. In addition to inflammatory cell infiltration, respiratory bronchus appears degeneration and necrosis. Capillaries appear dilation, congestion and bleeding in the infected group. Alveoli were suffered from rupture, dissolution and bronchial and alveolar epithelium appeared necrosis and were fell down from the lungs of infected mice. Collectively, similar to previous studies (Ren et al., 2013a; Chen et al., 2014), PmCQ2 infected mice have similar clinical and microscopical characters with what observed in cattle, indicating mice can serve as a surrogate model for studying *P. moltocida* infection.

### Evaluation of transcriptomic sequencing quality

After Illumina sequencing, there were approximately 5–7 × 10^7^ raw reads in two groups including three mice per group. The average numbers of clean reads in infected and control samples were 71442307 and 56323661, respectively. Thus, there were nearly more raw reads (10^7^) in the infected samples than control samples. The percentages of mapped to reference genome were more than 85% in all samples, in addition to 76.42% in only one infectious sample. Moreover, the percentage of GC content in all these samples was almost 50%. All the assessment criteria of base quality (Q30) were more than 94%. All data about quality of transcriptomic sequencing were shown in Supplementary Table 2.

### Functional analysis and classification of DEGs

**Figure 2:**
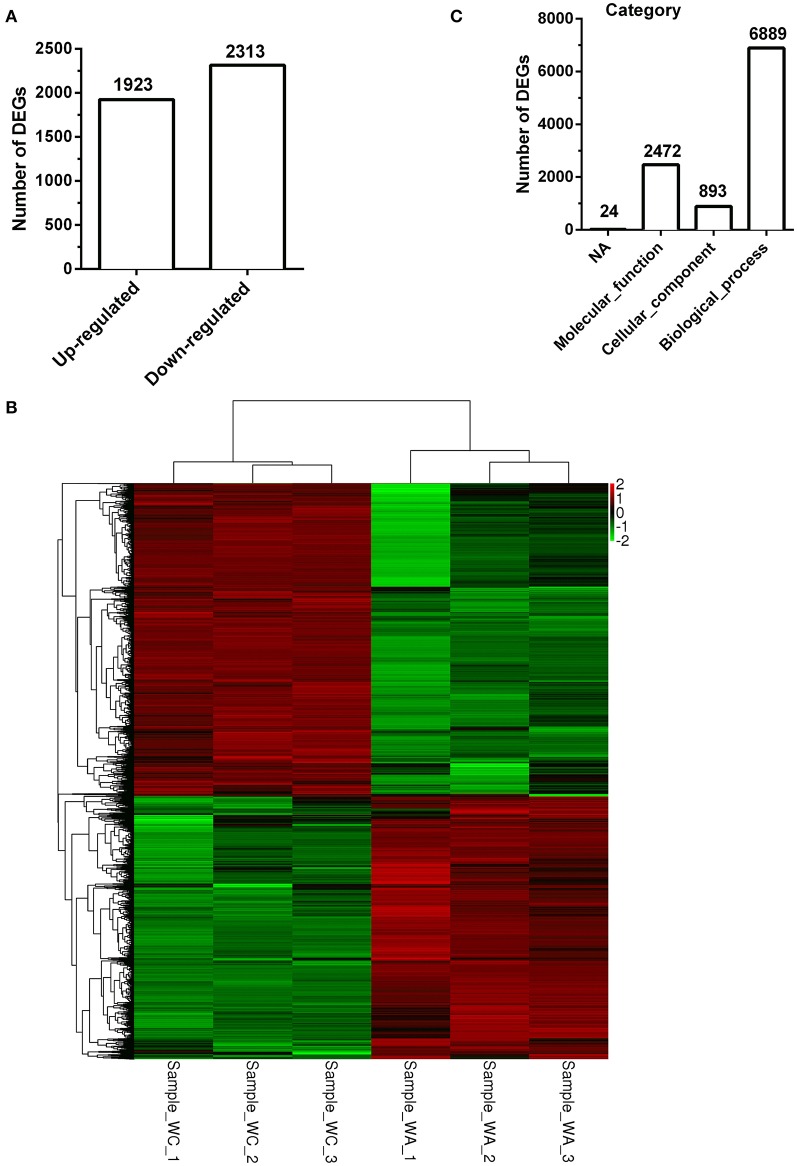
Clustering and category of DEGs in Illumina sequencing. **(A)** Numbers of up/down-regulated DEGs (*n* = 3). **(B)** The clustering of DEGs in heatmap. Hierarchical cluster analysis is used to identify DEGs with certain patterns of expression. The same sample can appear in the same cluster by the clustering. Sample_−_WC_−_1, Sample_−_WC_−_2 and Sample_−_WC_−_3 represent three duplication of control group, while Sample_−_WA_−_1, Sample_−_WA_−_2 and Sample_−_WA_−_3 represent three duplication of infected group. **(C)** The category of GO enrichments.

There were total 4236 DEGs between control and infected group with 1924 up-regulated DEGs (Figure 2A). The description of total DEGs was displayed in Supplementary Table 3. DEGs were used to unsupervised clustering hierarchy in this study. Correlation among samples was calculated by expression level of DEGs. In general, the same sample was classified into the same cluster by clustering analysis, and DEGs in the same cluster had lots of similar function, which was displayed with heatmap. Therefore, as shown in Figure 2B, three samples separately in control group or infected group had a tight correlation, whereas, there was almost no correlation between the two groups.

The function of DEGs was annotated by GO enrichments and KEGG enrichments. GO enrichments were divided into biological process (6889), cellular component (893) and molecular function (2,472) (Figure 2C). Total DEGs were annotated by 10278 GO terms of which 5303 GO terms were significant (*p* < 0.05) (Table 2). Up-regulated DEGs were significantly (*p* < 0.05) annotated to 4182 GO terms while down-regulated DEGs were 3540 GO terms (Table 2). In addition, DEGs were analyzed using KEGG database. Similarly, there were total 291 KEGG pathways and 116 KEGG pathways were significant (*p* < 0.05) (Table 3).

**Table 2:** GO annotation of DEGs

**Groups**	**Tested terms**	***P* < 0.05**	***P* < 0.01**
WC-vs.- WA-Down	7,063	3,540	1,667
WC-vs.- WA-Total	10,278	5,303	3,376
WC-vs.- WA-Up	7,198	4,182	2,739

**Table 3:** KEGG annotation of DEGs.

**Groups**	**Tested terms**	***P* < 0.05**	***P* < 0.01**
WC-vs.- WA-Down	274	74	52
WC-vs.- WA-Total	291	116	85
WC-vs.- WA-Up	276	97	75

To further analyze GO/KEGG enrichments of up/down-regulated DEGs, significance of GO/KEGG enrichment score (*p* < 0.05) and number of DEGs (greater than or equal to 2) were acted as a cutoff. There were 2195 GO enrichments in up-regulated DEGs and 1846 enrichments in down-regulated DEGs (Supplementary Tables 4, 5). Ten top GO enrichments in each functional ontology were showed in Supplementary Figure 1 and Figure 2. In up-regulated DEGs, the dominant GO terms of biological process were “positive regulation of interleukin-23 production” and “response to interferon-beta”; of cellular component were “Ndc80 complex” and “NLRP3 inflammasome complex”; and of molecular function were “C-C chemokine binding” and “interleukin-2 binding” (Supplementary Figure 1). In down-regulated DEGs, they were “positive regulation of integrin biosynthetic process” and “smoothened signaling pathway involved in regulation of cerebellar granule cell precursor cell proliferation” in biological process; “collagen type V trimer” and “fibrillar collagen trimer” in cellular component; and “hedgehog family protein binding” and “calcium ion binding” in molecular function (Supplementary Figure 2).

**Figure 3:**
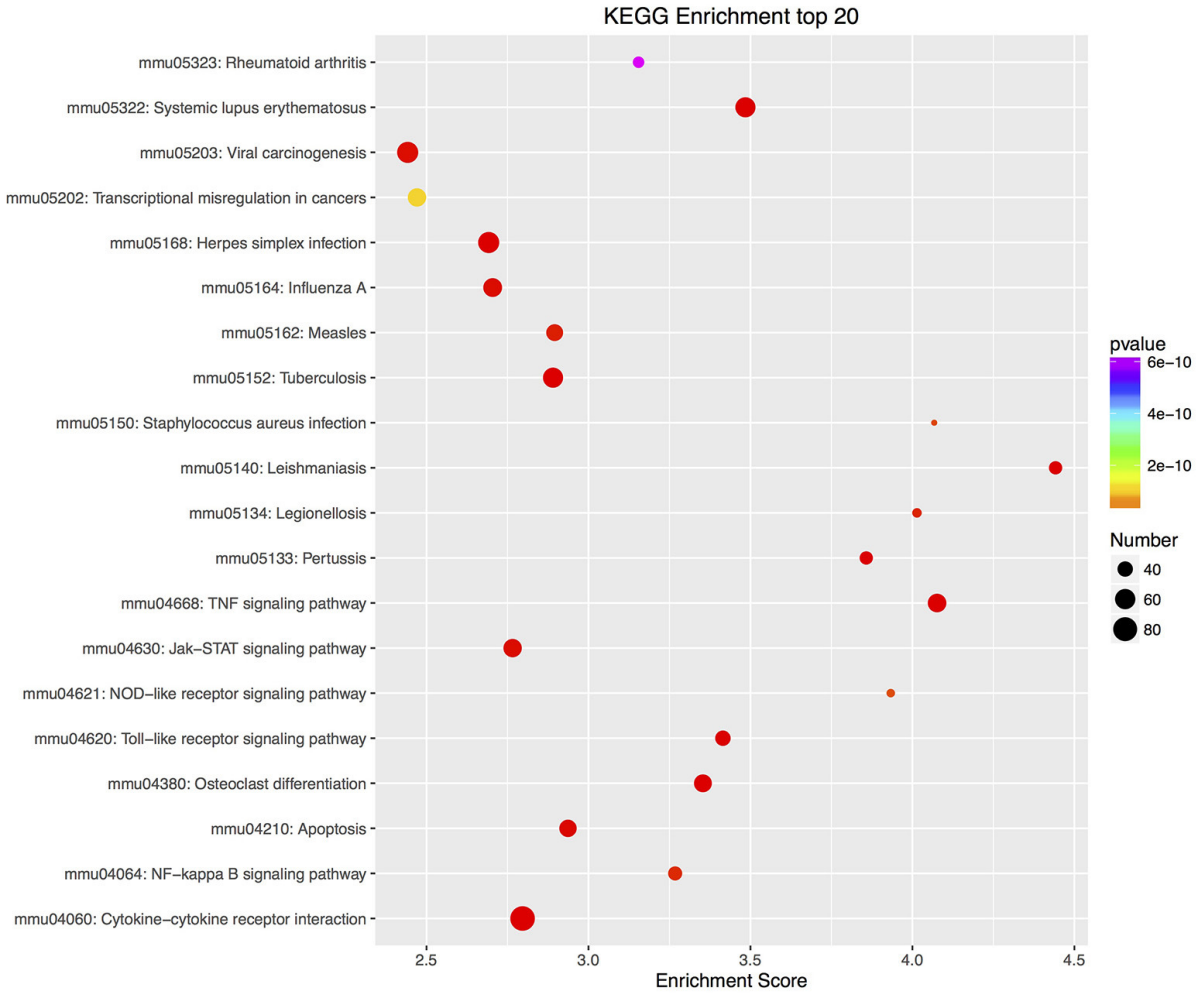
KEGG annotation of up-regulated DEGs. The numbers of DEGs in each pathway are counted and KEGG enrichment scores and *P*-values are acquired by hypergeometric distribution test. Top 20 KEGG enrichments with up-regulated DEGs larger than 2 were showed. The x-axis represents KEGG enrichment scores and the y –axis represents pathway terms. The colors of circle indicate *P*-values and the size of circle indicates the numbers of DEGs. The circle with redder and larger indicating that the enrichment of the pathway is higher and DEG number is larger in the pathway.

**Figure 4:**
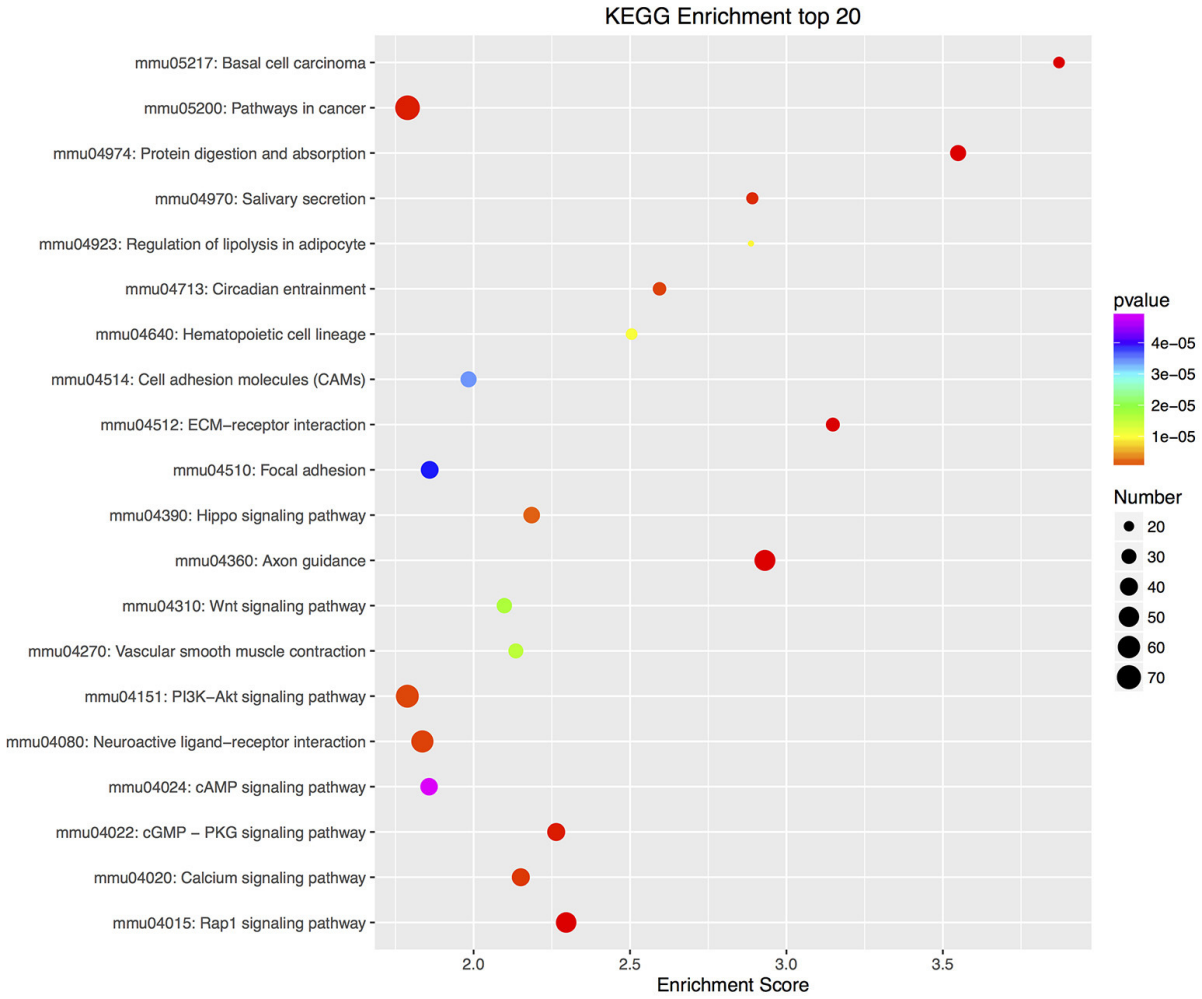
KEGG annotation of down-regulated DEGs. Similar to up-regulated DEGs, all down-regulated DEGs were mapped to signal pathway by KEGG database. DEGs numbers in each pathway were counted and KEGG enrichment scores and *P*-value were acquired by hypergeometric distribution test. Top 20 KEGG enrichments with down-regulated DEGs larger than 2 were showed. The x-axis represents KEGG enrichment scores and the y –axis represents pathway terms. The colors of circle indicate *P*-values and the size of circle indicates the numbers of DEGs. The circle with redder and larger indicating that the enrichment of the pathway is higher and DEG number is larger in the pathway.

Furthermore, there were 97 significant KEGG pathways in up-regulated DEGs and 74 in down-regulated DEGs (Supplementary Tables 6, 7). Twenty top KEGG enrichments in up/down-regulated DEGs were showed in Figures 3, 4, respectively. In 20 top KEGG enrichments of up-regulated DEGs, there were 7 KEGG pathways which may be involved in host responses against pathogens, namely, “TNF signaling pathway,” “Jak-STAT signaling pathway,” “NOD-like receptor signaling pathway,” “Toll-like receptor signaling pathway,” “Apoptosis,” “NF-kappa B signaling pathway,” and “Cytokine-cytokine receptor interaction” (Figure 3). While, in down-regulated DEGs, there were mainly pathways related to metabolism or autoregulation in 20 top KEGG enrichments (Figure 4), such as, “Protein digestion and absorption,” “Salivary secretion,” “Circadian entrainment,” “Regulation of lipolysis in adipocyte.”

### DEGs related to host immune responses against bacterial infection

DEGs related to immune responses were summarized via GO enrichments (*P* < 0.05) and listed in the Supplementary Table 8. There were 13 GO terms correlated with immune responses, including, “interferon-gamma-mediated signaling pathway,” “positive regulation of interleukin-23 production,” “response to interferon-beta,” “Bcl-2 family protein complex,” “NLRP3 inflammasome complex,” “Toll-like receptor 2-Toll-like receptor 6 protein complex,” “interleukin-2 binding,” “Fc-gamma receptor signaling pathway,” “positive regulation of natural killer cell chemotaxis,” “chemokine activity,” “cytokine activity,” “response to molecule of bacterial origin,” and “T cell differentiation involved in immune response.” The 13 GO terms included 158 DEGs in total and in which there were only 26 down-regulated DEGs (16%). These GO terms were mainly involved in pattern recognition receptor (PRR), apoptosis, chemokine, inflammatory cytokines and T cell differentiation, which were involved in resistant to bacterial infection.

In addition, biosynthetic or signal transduction pathways about defense response via KEGG annotation were significantly enriched. There were 20 KEGG pathways in up-regulated DEGs, while only 7 KEGG pathways in down-regulated DEGs involved in immune responses (Supplementary Tables 9, 10). By using DEGs number with greater than 50 in these KEGG pathways as a cutoff, up-regulated DEGs were mapped to 4 KEGG pathways, including “Cytokine-cytokine receptor interaction,” “PI3K-Akt signaling pathway,” “TNF signaling pathway,” and “Jak-STAT signaling pathway,” while down-regulated DEGs were mapped to “PI3K-Akt signaling pathway” and “Rap1 signaling pathway.”

### Experimental verification of DEGs

By comprehensive analysis of GO and KEGG annotation of DEGs, numerous DEGs in IL-12/IFN-γ axis and IL-23/IL-17 axis were found (Supplementary Table 11). The previous study reported that in pulmonary inflammation, IL-23 or IL-6 and transforming growth factor β (TGF-β) synergistically induced differentiation of naïve CD4^+^ cells into Th17 cells, favoring proliferation of memory T-cells and production of IL-17 (IL-17A) and IL-17F, associating with the STAT3 (signal transducers and activators of transcription-3) signaling (Iwakura and Ishigame, 2006; Mangan et al., 2006; Aujla et al., 2007; Wilson et al., 2007; Vanaudenaerde et al., 2008). IL12 is an important regulatory cytokine in Th1 responses through inducing the differentiation of Th1 cells to produce interferon (IFN-γ), associating with STAT1 signaling (Trinchieri, 2003; Qing and Stark, 2004; Iwakura and Ishigame, 2006). Interestingly, the key components of IL-23/IL-17 axis, including IL-23, IL-6, TGF-β, IL-17, IL-17F, IL-17 receptor, and STAT3, were significantly increased in transcriptional level and the key components of IL-12/IFN-γ axis, including IL12a/b (p35/p40), IFN-γ, IFN-γ receptor, JAK2, and STAT, were also all significantly increased (Supplementary Table 11).

**Figure 5:**
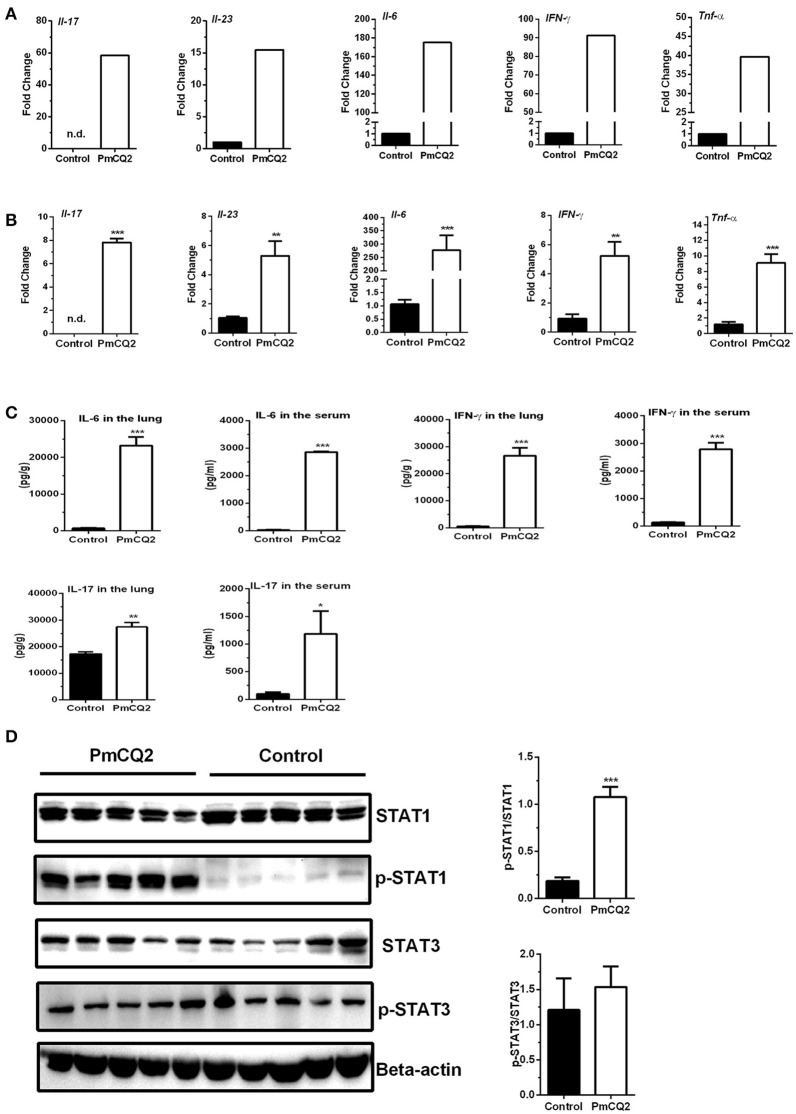
Up-regulation of genes related to *Ifn-*γ*/Il-17* at 16 hpi. **(A)** The high expression of *Ifn-*γ and *Il-17* after *P. multocida* infection by Illumina sequencing (*n* = 3). The fold change indicated the ratio of the experimental group basemean (sample-WA average expression level) to the control group basemean (sample-WC average expression level) by Illumina sequencing. **(B)** The high expression of *Ifn-*γ and *Il-17* after *P. multocida* infection by qRT-PCR (*n* = 6, unpaired, two-tailed Student's *t*-test). **(C)** The levels of IFN-γ and IL-17 in the lungs or serum using ELISA analysis (*n* = 6, unpaired, two-tailed Student's *t*-test). **(D)** The abundance of p-STAT1^Y701^ and p-STAT3^Y705^ in the murine lungs by immunoblotting analysis (*n* = 5, unpaired, two-tailed Student's *t*-test). All data were expressed as means ±SEM. Because *Il17* was not detected in control mice, the fold changes of *Il17* in treatment mice were showed via presuming expression quantity = 1 of control mice in RNA-Seq **(A)** and Ct = 30 in qRT-PCR **(B)**, respectively. ^*^P < 0.05, ^**^P < 0.01, ^***^P < 0.001.

To validate the reliability of transcriptomic sequencing, inflammatory cytokines in IL-12/IFN-γ axis and IL-23/IL-17 axis were selected to detect by qRT-PCR. Figures 5A,B showed expression level of DEGs (*Il-6, Il-23, Il-17, IFN-*γ*, and Tnf-*α*)* by RNA-seq and qRT-PCR, respectively. Similar to that observed with RNA-seq analysis, they are found to significantly increase by qRT-PCR analysis (Figures 5A,B). To further confirm post-translational level of these DEGs *in vivo*, levels of IL-6, IL-17, and IFN-γ in the lungs or serum were determined using ELISA kits, and activation of STAT1 and STAT3 signaling were analyzed with immunoblotting. We found that levels of IL-6, IL-17, and IFN-γ in the lungs or serum in infectious mice were significantly higher than that in control mice (Figure 5C). There was no difference in the abundance of total STAT1 in the lungs between infectious mice and control mice, whereas, abundance of p-STAT1 in the lungs of infectious mice were significantly increased, compared to control group (Figure 5D). While the abundance of total STAT3 and p-STAT3 were similar between two groups (Figure 5D).

## Discussion

*P. multocida* serogroup A is an opportunistic pathogen causing severe respiratory diseases in cattle (Rerat et al., 2012; Li et al., 2016). In this study, a mouse model of *Pasteurella* pneumonia caused by PmCQ2 was established via intraperitoneal infection. It is well known that the natural infectious route for *P. multocida* serotype A strain is through intranasal infection, but we found that intranasal infection results in a poor reproducibility among mice. Moreover, there are some similarities between intranasal infection and intraperitoneal infection, including the pathological alterations and expressions of inflammatory cytokines (Praveena et al., 2010; Hodgson et al., 2013). Thus, intraperitoneal infection was used in this study. The mice died within 3 days post-infected by PmCQ2. This result indicates that PmCQ2 is a virulent strain for mice as reported previously (Li et al., 2016). Likewise, this study indicates that PmCQ2 also proliferates significantly in the lungs of mice after inoculation, but the bacterial burden tends to be stable after 16 h post-infection from previous observation (Li et al., 2016). However, in current study, the bacterial load increases after 16 h post-infection by bacterial counting analysis. Traditional counting bacteria by gradient dilution cannot display detailed distribution of bacteria (Amann et al., 1995). Thus, the bacterial load in the lungs was also detected with FISH using an oligonucleotide probe pmhyb449, which recognizes specifically 16S rRNA of *P. multocida* (Mbuthia et al., 2001). The conclusion from FISH analysis is similar with the previous investigation (Li et al., 2016). It is worth noting that bacterial load after 24 h post-infection from FISH is not consistent to that from gradient dilution. We suspect this difference may be caused by limitations of the number of lung sections in FISH. *Pasteurella* pneumonia in cattle, rabbits, fowl and pigs has been reported and is mainly caused by *P. multocida* serogroup A (Blackall et al., 1995; Al-Haddawi et al., 2000; Pors et al., 2011; Praveena et al., 2014). A previous study reported that *P. multocida* causes inflammation because of neutrophil and macrophage infiltration in the lungs of cattle by intratracheal route (Praveena et al., 2014). In this study, like other *P. multocida* serogroup A, PmCQ2 causes serious lung lesions in mice characterized by inflammation in murine lungs. This suggests that there is a similar inflammatory response in murine lungs after PmCQ2 infection by intraperitoneal routes. However, the cell type in the lungs was not further confirmed by other methods, such as flow cytometry and IHC. In addition, the infected tissue section shows that there are different degrees of bronchitis, alveolar rupture and some hemorrhage, which are consistent to pathological changes in cattle infected by *P. multocida* (Mathy et al., 2002; Dabo et al., 2007). However, the underlying mechanism of host inflammatory responses to this bacterial infection is still elusive. Thus, the DEGs related to *P. multocida* infection are explored in this study with transcriptomic sequencing because it can provide a wide range of changes in transcription level. In this study, we mainly studied the immune responses in mice against *P. multocida*, such as, PRRs, downstream pathways of PRRs and effector molecules (e.g., cytokines and chemokines).

PRRs are crucial for the initiation of innate immunity against bacterial infection, via recognizing pathogen-associated molecular patterns (PAMPs) (Jang et al., 2015). Retinoic acid-inducible gene-I (RIG-I)-like receptors (RLRs), toll-like receptors (TLRs), C-type lectin receptors (CLRs), AIM2-like receptors (ALRs) and nucleotide-binding oligomerization domain (NOD)-like receptors (NLRs) are from PRR families (Kawai and Akira, 2009; Jang et al., 2015). Activation of TLRs, except for TLR3, recruits the adaptor protein myeloid differentiation factor 88 (MyD88) to form receptor complex (Kawai and Akira, 2005). NOD1, NOD2, and NLRP3 (NOD-like receptor family, pyrin domain containing, NLRP) are important components of NLR families. NOD1, NOD2, TLRs/MyD88 complex stimulate nuclear factor kappa B (NF-κB) signaling and mitogen activated protein kinase (MAPK) signaling against bacteria. NLRP3 inflammasome consists of NLRP3, the adaptor apoptosis-associated speck-like protein containing a CARD (ASC) and the effector caspase-1 (Levy et al., 2015). Activation of NLRP3 inflammasome leads to secretion of mature IL-18/IL-1β (Kanneganti et al., 2007).

In this study, there were various DEGs involved in “Toll-like receptor signaling pathway,” “RIG-I-like receptor signaling pathway,” and “NOD-like receptor signaling pathway” by KEGG annotation (Supplementary Table 12). In “Toll-like receptor signaling pathway,” more than 90.9% of DEGs are up-regulated, including *Tlr1, Tlr2, Tlr6, Tlr9*, and *MyD88*. TLR2 functions as a homodimer or as a heterodimer with TLR1 or TLR6 to recognize a variety of PAMPs (Li and Sun, 2016). TLR2/TLR1 recognizes triacylated lipoproteins/lipopeptides while TLR2/TLR6 recognizes diacylated lipoproteins/lipopeptides (Schenk et al., 2009). TLR9 recognizes CpG DNA in bacterial genomes (Medzhitov, 2007). To date, various investigations have reported the involvement of TLRs during infections, such as *Sporothrix schenckii* (TLR2), *Staphylococcus aureus* (TLR2), *Salmonella enterica* (TLR1/TLR2 complex, TLR9), *Vibrio cholerae* cytolysin (TLR2/TLR6 complex, TLR9) (Totemeyer et al., 2005; de C Negrini et al., 2014; Khilwani et al., 2015; Nandi and Bishayi, 2015). However, as far as we known, the association of TLRs during *P. multocida* infection is mainly about TLR4 to recognize *P. multocida* toxin (PMT) and LPS (Hildebrand et al., 2012; Kubatzky, 2012). Our results indicate that other TLRs, especially, TLR2, mediate *P. multocida* infection. *Nod1, Nod2*, and *Nlrp3* significantly up-regulate from transcriptional level. NOD1 recognizes γ-D-glutamyl-meso-diaminopimelic acid (iE-DAP) mainly from gram-negative bacteria, while NOD2 is a general sensor of peptidoglycan through the recognition of muramyl dipeptide (MDP) (Girardin et al., 2003). Like TLR induction, *Shigella flexneri*, entero-invasive *Escherichia coli, Chlamydophila pneumoniae, Campylobacter jejuni*, and *Listeria monocytogenes* are sensed by NOD1, while *Streptococcus pneumonia* and *Mycobacterium tuberculosis* are recognized by NOD2 (Kanneganti et al., 2007). Therefore, we presume that NLRs are also involved in the *P. multocida* infection.

As downstream pathways of PRRs, NF-κB, and MAPK signaling pathways are enriched by KEGG annotation. There are total 82 DEGs involved in MAPK signal pathway and 45 DEGs in NF-κB signaling. NF-κB and MAPK signaling play important roles in defense responses against bacterial infection by regulating production of cytokines, such as *Enterococcus faecalis, S. aureus*, and *M. tuberculosis* (Zhu et al., 2013; Deng et al., 2014; Zou and Shankar, 2015). Thus, the NF-κB and MAPK signaling may also have the critical importance during *P. multocida* infection. *Nlrp3, caspase-1*, and *Pycard* up-regulate significantly, which are involved in assembling of NLRP3 inflammasome to promote maturation of pro-IL18/IL-1β. Its activation contributes to host defense during a variety of bacterial infections, suggesting that it is involved in defense against *P. multocida* infection. Indeed, we have found that *Nlrp3* is significantly up-regulated in RAW264.7 cells after *P. multocida* infection (data unpublished).

During the development of inflammation, cytokines play important roles, mainly embodying as restriction and balance between proinflammatory and anti-inflammatory cytokines. Cytokines have also key functions in host defense responses against bacteria. Some cytokines (e.g., IL-1) are acted as potential useful biomarkers of community-acquired bacterial infection (Holub et al., 2013). There are various DEGs involved in cytokine mediating signal pathway by KEGG enrichments during *P. multocida* infection (Supplementary Table 13). 117 DEGs are included in cytokine-cytokine receptor interaction, and in which 84 DEGs are up-regulated. The type of up-regulated cytokines (e.g., *Ifn-*γ, *Tnf-*α, *Il-1*β, *Il-6*) in this study has some similarity to that of bovine lung infected by *P. multocida* (Mathy et al., 2002), which suggests that in addition to similar pathological changes, immunological changes in mice may be also similar to those in cattle caused by *P. multocida*. However, a further study is needed to further explore the similarity and difference in responses to *P. multocida* in mice and cattle.

Interestingly, various up-regulated cytokines is involved in Th17 responses. In this study, although p-STAT3 is not significantly increased, Th17 related cytokines (*Il-17, Il-17ra, Il-23a, Il-6, and Il-6r*α) are significantly increased using transcriptomic sequencing. Moreover, increased expressions of *Il-6, Il-17*, and *Il-23* are validated by qRT-PCR. Also, the levels of IL-17 and IL-6 are significantly increased in the pulmonary homogenate and serum at 16 h post-infection. It has been reported that host immune responses are mainly Th17 responses in pulmonary inflammation caused by bacteria (Caucheteux et al., 2016; Hua et al., 2016; Wong et al., 2016). For example, in pulmonary inflammation, IL-23 is secreted mainly by alveolar dendritic cells (DCs) and macrophages to induce the differentiation of naïve CD4 cells into Th17 cells for production of IL-17 (Iwakura and Ishigame, 2006; Aujla et al., 2007; Caucheteux et al., 2016). In addition, IL-6 and TGF-β synergistically stimulates the production of IL-17 (Mangan et al., 2006; Wilson et al., 2007). This process is regulated mainly by transcription factors Foxp3, RORγt (retinoid-related orphan receptor), STAT3 and IRF4 (interferon regulatory factor-4) (Vanaudenaerde et al., 2008; Voo et al., 2009). IL-17 could recruit neutrophils into lung to eliminate the invasive bacteria through granulopoiesis and CXC chemokines induction (Kolls and Linden, 2004). Release of IL-17 also induces antimicrobial proteins and proinflammatory cytokines, including granulocyte colony-stimulating factor (G-CSF), IL-6, IL-1β, and TNF-α and hematopoietic growth factor (Jovanovic et al., 1998; Happel et al., 2005; Dubin and Kolls, 2007). Thus, IL-23/IL-17 axis plays a critical role in anti-bacterial immunity, such as *Pseudomonas aeruginosa, Citrobacter rodentium, E. coli, Porphyromonas gingivalis* (Mangan et al., 2006; Dubin and Kolls, 2007; Shibata et al., 2007; Yu et al., 2008; Ren et al., 2017). The results from current study indicate like other reported bacteria, Th17 responses is also involved in *P. multocida* infection by an intraperitoneal route. In fact, it has been reported that PMT induces the differentiation of Th17 cells through the activation of STAT transcription factors to induce IL-17 production (Hildebrand et al., 2015), which suggest IL-17 is involved in *P. multocida* infection. Notably, intranasal immunization in mice protects mice against multiple bacterial pneumonia (e.g., *P. aeruginosa, S. pneumonia*) in an antibody-independent but IL-17–dependent manner (Wu et al., 2012; Wang et al., 2017), which suggest the feasibility of development a broadly protective vaccine against bacterial pneumonia by targeting lung Th17 cells. Thus, it is interesting to know whether the manipulation of lung Th17 responses could regulate *P. multocida* infection or not.

In this study, Th1 related cytokines (*Il-12a, Il-12b, Il-12r*β*1, Il-12r*β*2, Ifn-*γ*, Tnf-*α*, Il-2r*α*, Il-2r*γ*, Il-2r*β) are up-regulated from transcriptomic sequencing, qRT-PCR, and ELISA analysis. Moreover, increased activation of p-STAT1, the regulator of Th1, is also observed. These results indicate that there are strong Th1 immune responses in mice during *P. multocida* infection. IL-12 is structural similar to IL-23 with the same IL-12p40 subunit and unique IL-12p35, and is an important regulatory cytokine in Th1 responses by inducing the differentiation of naïve CD4 cells into Th1 cells and activating natural killer cells for the production of IFN-γ (Trinchieri, 2003; Happel et al., 2005; Iwakura and Ishigame, 2006). IFN-γ activates both neutrophils and macrophages for intracellular killing of bacteria or fungi (Nguyen et al., 2015). The process of IFN-γ or IFN regulatory factor-1 (IRF-1) production is regulated by STAT1 (Qing and Stark, 2004; Iwakura and Ishigame, 2006). In return, recognition of IFN-γ by IFN-γ receptor (IFNGR1/2) activates Janus kinase (JAK, mainly JAK1 and JAK2)/STAT1 pathway to activate transcription factor T-bet, resulting in extensive production of IFN-γ to eliminate virus or bacteria (Rawlings et al., 2004; Hu and Ivashkiv, 2009). Thus, IL-12/IFN-γ axis has critical importance in lung infection. IL-12 is important in the initial phase of bacterial, parasitic, and viral infections (Tekkanat et al., 2001). Recently, a study indicated that IL-12 plays a critical role in protection from pulmonary methicillin-resistant *S. aureus* (MRSA) via activating IL-12/ IFN-γ axis (Nguyen et al., 2015). In addition, IL-12 promotes IFN-γ-dependent neutrophil recruitment into the lungs of mice after intranasal infection with *S. pneumonia* (Sun et al., 2007). Specifically, IL-12 and IFN-γ induce synergistically bacterial clearance from the lungs and pulmonary cellular infiltration during extracellular and intracellular bacterial infection, such as *L. monocytogenes, S. pneumonia, Chlamydia muridarum, M. tuberculosis* (Haring et al., 2005; Mpiga and Ravaoarinoro, 2006; Sun et al., 2007; Cooper and Khader, 2008; Jupelli et al., 2010; Hashiguchi et al., 2014). More importantly, *Il-12a, Il-12b, Il-2, Ifn-*γ are up-regulated in bovine lungs by an intratracheal *P. multocida* inoculation (Mathy et al., 2002), which suggest that Th1 responses are induced by *P. multocida* and there are some similarities between mice and cattle during *P. multocida* infection by an intratracheal or intraperitoneal route. However, Th1 responses in host during *P. multocida* infection with an intranasal route still need to further investigation.

In addition to cytokine mediating signal pathway, chemokine signaling is also a significant signal pathway from KEGG annotation. Based on cysteine residues position, chemokines are classified into two major chemokine sub-families, CXC chemokines and CC chemokines. The former is mainly chemotactic for neutrophils and the latter mainly hemotactic for monocytes and sub-set of lymphocytes (Palomino and Marti, 2015). In this study, there are 14 CC DEGs, including, *Ccl3, Ccl4, Ccl9, Ccl11, Ccl22, Ccl17, Ccl5, Ccl12, Ccl7, Ccl2, Ccl19, Ccl28, Ccl27a*, and *Ccl21a*. Except for the last two, others are up regulated. In addition, 8 CXC DEGs are up regulated, including *Cxcl16, Cxcl14, Cxcl13, Cxcl5, Cxcl19, Cxcl9, Cxcl10*, and *Cxcl2*. Only *Cxcl12* decreases significantly in transcriptional level. Tagawa et al. showed CC chemokines induces macrophage trafficking to the site of infection during *E. coli* infection (Tagawa et al., 2004). Abundant macrophage inflammatory protein 1α (MIP-1α, namely CCL3) is detected in the liver during *L. monocytogenes* (Bubonja et al., 2006). Similarly, MIP-1α, MIP-1β, and RANTES (namely CCL3, CCL4, and CCL5) play key roles in *Helicobacter pylori* (Raghavan et al., 2003). Moreover, CXC chemokines are involved in various bacterial infections by impacting neutrophils, for example, *P. aeruginosa, M. tuberculosis* (Almeida et al., 2009; Gregson et al., 2013). In this study, we found infiltrated cells in the lungs are mainly the neutrophils, with lower number of macrophages in pulmonary lesion, suggesting that CXC chemokines have critical roles during *P. multocida* infection. However, the involvement of neutrophils, macrophages or CXC chemokines in *P. multocida* infection needs further investigation.

In conclusion, a mouse model of *Pasteurella* pneumonia is established successfully by *P. multocida* serogroup A strain PmCQ2 in this study. This study firstly showed that there is significant alteration in expression profile in the lungs during *P. multocida* infection *in vivo* using transcriptomic sequencing. Most of altered genes are correlated to PRRs, PRR downstream signaling, chemokines and cytokines, indicating the complex immune responses in the lungs during *P. multocida* infection. This study provides a wide range of new insights to defense responses during *P. multocida* infection.

## Author contributions

YP, NL, and WR conceived the experiments. CW and XQ conducted the experiments. XQ, PL, and TP contributed to data analysis. CW and WR wrote the paper. YP, NL, and WR supervised the project and revised the paper.

### Conflict of interest statement

The authors declare that the research was conducted in the absence of any commercial or financial relationships that could be construed as a potential conflict of interest.

## Supplementary material

Supplementary Figure 1 GO analysis of up-regulated DEGs. All up-regulated DEGs were analyzed by GO enrichments and numbers of DEGs in each GO term were counted. Significance of GO enrichments was described as *P* < 0.05. In this figure, top 10 GO enrichments with up-regulated DEGs larger than 2 in each functional ontologies were showed.

Supplementary Figure 2 GO analysis of down-regulated DEGs. All down-regulated DEGs were analyzed by GO enrichments and numbers of DEGs in each GO term were counted. Significance of GO enrichments was described as *P* < 0.05. In this figure, top 10 GO enrichments with down-regulated DEGs larger than 2 in each functional ontologies were showed.

Supplementary Table 1 Primers used in qRT-PCR analysis.

Supplementary Table 2 RNA-seq sequencing evaluation.

Supplementary Table 3 Total DEGs in this study.

Supplementary Table 4 Significant GO enrichment analysis of up-regulated DEGs.

Supplementary Table 5 Significant GO enrichment analysis of down-regulated DEGs.

Supplementary Table 6 Significant KEGG pathways of up-regulated DEGs.

Supplementary Table 7 Significant KEGG pathway of down-regulated DEGs.

Supplementary Table 8 DEGs related to immune response.

Supplementary Table 9 KEGG pathway of up-regulated DEGs related to immune response.

Supplementary Table 10 KEGG pathway of down-regulated DEGs related to immune response.

Supplementary Table 11 Gene list of significant DEGs in IL-12/IFN-γ axis and IL23/IL17 axis.

Supplementary Table 12 Expression level of DEGs related to PRR and PRR downstream signaling.

Supplementary Table 13 Expression level of DEGs related to cytokine and chemokine signaling.

## Abbreviations

ALRs: AIM2-like receptor
ASC: apoptosis-associated speck-like protein containing a CARD
BCA: bicinchoninic acid
CLRs: C-type lectin receptors
CFU: colony-forming units
DEGs: differently expressed genes
DAPI, 4′: 6-diamidino-2-phenylindole
ELISA: enzyme linked immunosorbent assay
FPKM: per million fragments mapped
FC: fold-change
FISH: fluorescence *in situ* hybridization
GO: gene ontology
G-CSF: granulocyte colony-stimulating factor
H&E: hematoxylin and eosin
iE-DAP: γ-D-glutamyl-meso-diaminopimelic acid
IFN-γ: interferon
IRF4: interferonregulatoryfactor-4
LPS: lipopolysaccharide
MAPK: mitogen activated protein kinase
MDP: muramyl dipeptide
MIP-1α: macrophage inflammatory protein 1α
MyD88: myeloid differentiation factor 88
NLRP: NOD-like receptor family, pyrin domain containing
NLRs: nucleotide-binding oligomerization domain (NOD)-like receptors
NF-κB: nuclear factor kappa B
PMT: *pasteurella multocida* toxin
PAMPs: pathogen-associated molecular patterns
qRT-PCR: quantitative real-time RT-PCR
KEGG: kyoto encyclopedia of genes and genomes
RLRs: retinoic acid-inducible gene-I (RIG-I)-like receptors
RORγt: retinoid-related orphan receptor
ROS: reactive oxygen species
STAT3: signal transducers and activators of transcription-3
SSC: saline-sodium citrate
SEM: standard error of mean
TLRs: toll-like receptors
TGF-β: transforming growth factor β.
