# Supplementary figures and images for: Transcriptomic Analysis on Responses of Murine Lungs to Pasteurella multocida Infection
Source: Front Cell Infect Microbiol. 2017 Jun 20;7:251. doi: 10.3389/fcimb.2017.00251 (PMC5476747; doi:10.3389/fcimb.2017.00251)

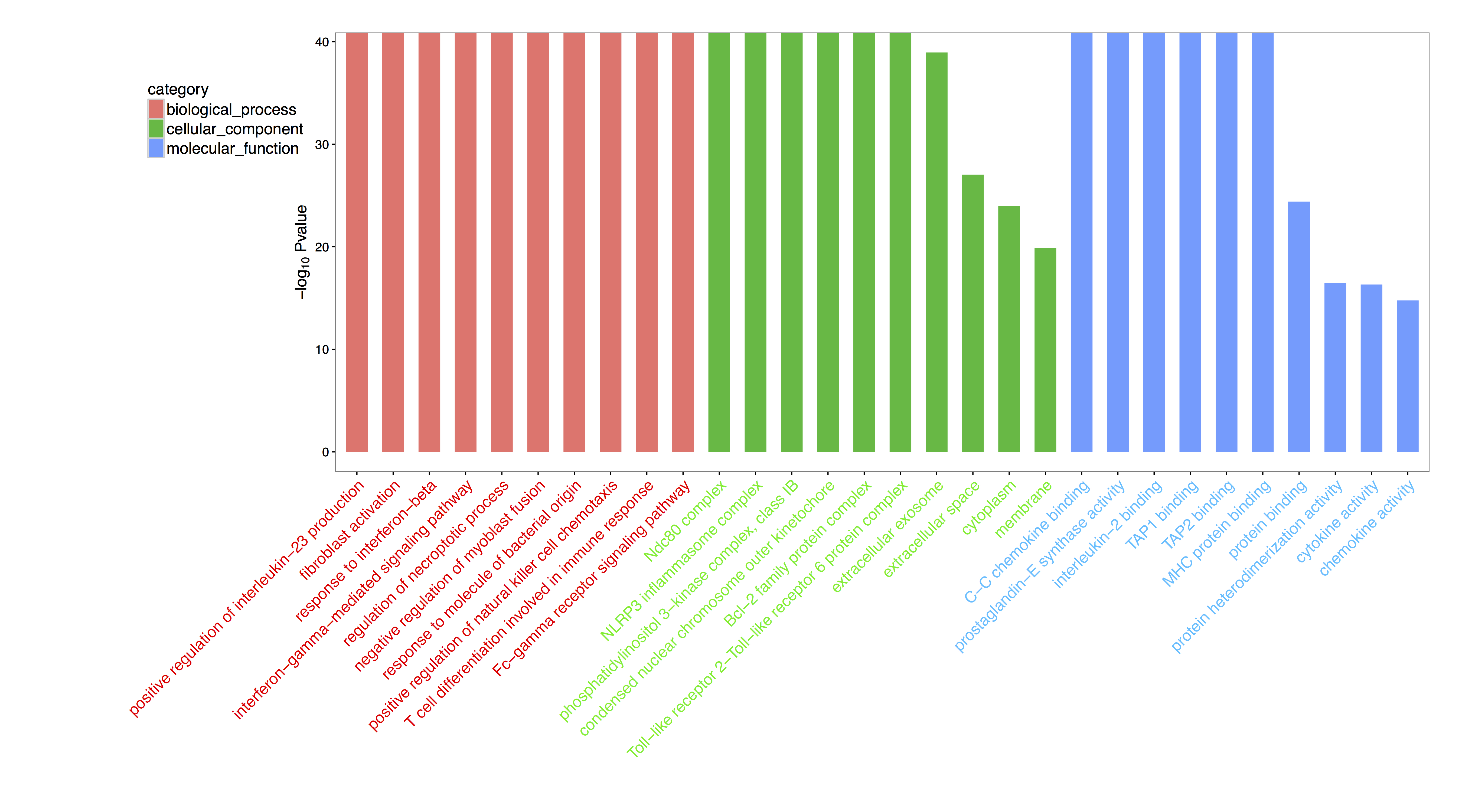

Supplement: Supplementary Figure 1 — GO analysis of up-regulated DEGs. All up-regulated DEGs were analyzed by GO enrichments and numbers of DEGs in each GO term were counted. Significance of GO enrichments was described as P < 0.05. In this figure, top 10 GO enrichments with up-regulated DEGs larger than 2 in each functional ontologies were showed. [file Image1.JPEG]

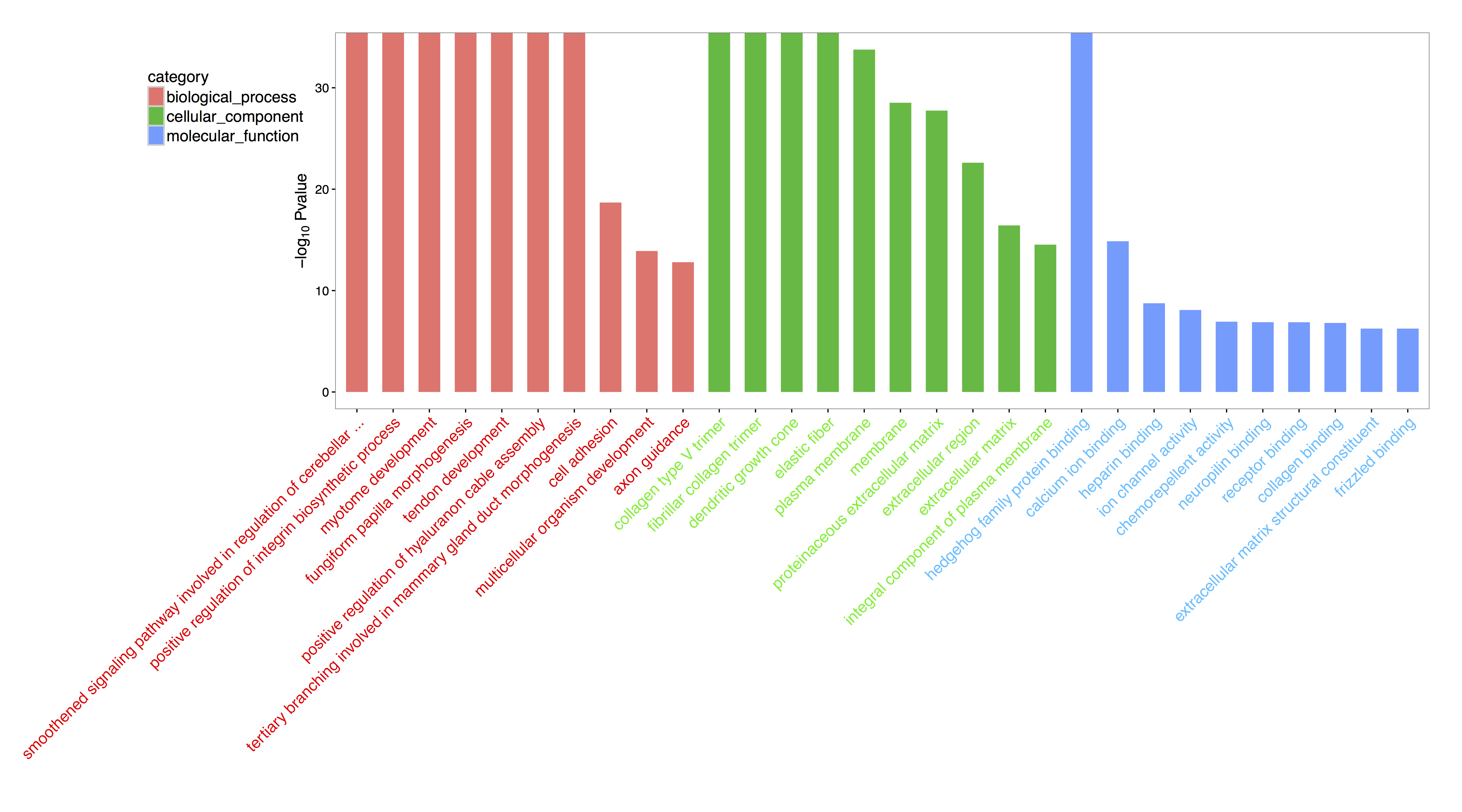

Supplement: Supplementary Figure 2 — GO analysis of down-regulated DEGs. All down-regulated DEGs were analyzed by GO enrichments and numbers of DEGs in each GO term were counted. Significance of GO enrichments was described as P < 0.05. In this figure, top 10 GO enrichments with down-regulated DEGs larger than 2 in each functional ontologies were showed. [file Image2.JPEG]
